# Controlling the Intrinsic Charge Carrier Properties of Two-Dimensional Monochalcogenides (GeSe)

**DOI:** 10.1021/acsomega.4c04343

**Published:** 2024-10-14

**Authors:** Defne Akay, Muhammed Batuhan Kocak

**Affiliations:** † Department of Physics, Faculty of Science, Ankara University, Ankara 06100, Turkey; ‡ Graduate School of Natural and Applied Science, Ankara University, Ankara 06110, Turkey

## Abstract

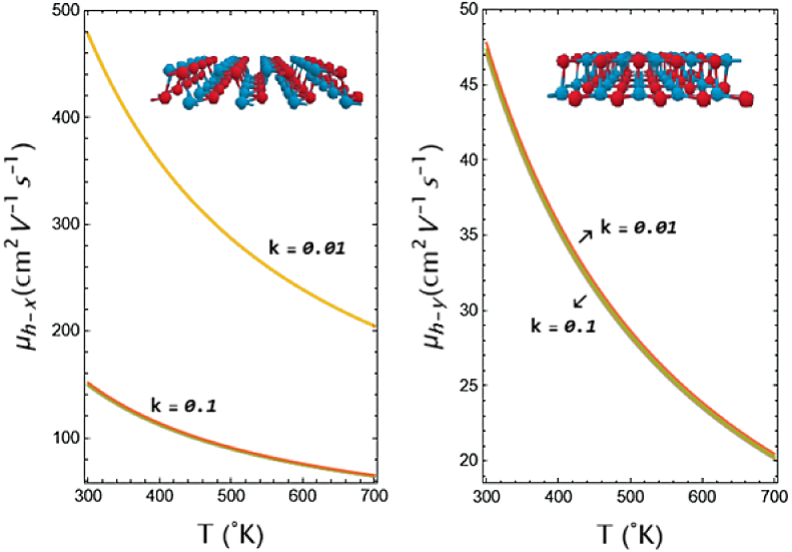

Strong anisotropy exhibited by materials, particularly in their low-dimensional forms, is a highly intriguing characteristic. In this study, we investigate the effects of geometrical potential and thermodynamics on the electronic properties of monolayer monochalcogenide charge carriers. First, the geometrical potential is introduced in a monolayer structure. We discuss the Fermi surface topology of materials and the effects of the geometrical potential on the low energy bands of 2D group-IV monochalcogenides around the Γ-point. Then, the temperature dependence of the carrier mobility of the monolayer is discussed along with predictions for its potential applications as nanomaterials.

## 1 Introduction

Two-dimensional (2D) semiconductors with a suitable band gap have broad potential applications in electronics and optoelectronics, including transistors, energy converters, and photodetectors.^1−3^ Graphene’s ultrahigh carrier mobility has the potential to revolutionize microelectronics and could potentially replace silicon. It is important to note that further research and development are needed to fully understand the capabilities and limitations of this material. This breakthrough has not only paved the way for further research on graphene itself but also sparked great enthusiasm for the synthesis of other two-dimensional (2D) structures of various inorganic layered materials. One such material is hexagonal boron nitride (h-BN),^4,5^ which has garnered considerable attention due to its unique properties and potential applications. Inspired by the success achieved with graphene, researchers have been actively exploring methods to synthesize 2D h-BN structures. Transition metal oxides have also been a focus of interest in the synthesis of 2D structures.^6^ These materials exhibit diverse properties and have applications in fields such as energy storage, catalysis, and electronics. By isolating and studying their 2D forms, scientists hope to unlock new possible applications for these materials. Graphitic nitrides,^7^ another class of inorganic layered materials, have also attracted attention in recent years. These compounds possess intriguing electronic and optical properties, making them promising candidates for various technological applications. Transition metal dichalcogenides (TMDs) are yet another group of materials that have been extensively studied in the quest of 2D structures.^8−10^ These compounds, composed of transition metals and chalcogen elements, exhibit unique electronic, optical, and mechanical properties. The successful synthesis of 2D TMD structures could open up new avenues for applications in electronics and energy conversion. The exploration of hexagonal boron nitride, transition metal oxides, graphitic nitrides, and transition metal dichalcogenides is just the beginning of a fascinating journey toward unlocking the potential of these materials in various technological applications. Currently, the most popular 2D semiconductors that have been isolated break either inversion symmetry, including BN and MoS_2_, or rotational symmetry, including black phosphorus^11−14^ and rhenium disulfide (ReS_2_)^15−17^, but not both. In recent years, the monolayer form of group IV monochalcogenides is predicted to be stable with the orthorhombic structure of black phosphorus.^18,19^ The 2D monochalcogenide structures with a folded structure similar to that of black phosphorus have attracted considerable attention.^20−24^ Group-IV monochalcogenides have been considered as potential absorber materials for solar energy conversion technologies.^25^ Among them, monolayer GeS is an indirect bandgap semiconductor with a high thermodynamic stability which is a highly anisotropic behavior,^26−28^ and GeSe is a direct bandgap monolayer structure and is expected to be an ideal candidate for nanoelectronic devices. Previous studies have shown that the electron mobility of monolayer monochalcogenides by traditional deformation potential theory (DPT) based on a longitudinal acoustic branch is widely used to predict the intrinsic mobility of carriers because of its simple theoretical formula, including three parameters of effective mass, elastic modulus, and deformation potential.^29−33^ The intrinsic carrier mobility is ultimately obtained from using first-principles calculations and Wannier function interpolation. The intrinsic mobility is then calculated using electron–phonon coupling (EPC) matrix elements using density functional theory (DFT) computations.^34,35^ It has been found that hole mobility is largely determined by longitudinal acoustic phonon scattering, while electron mobility is mainly dominated by optical phonon scattering.^36^ Additionally, the synergistic effect of the presence of 2D occupied antibonding states in the materials and band alignment at the interfaces can also affect the electronic properties.^200^ Overall, this is the range of predicted carrier mobilities for monolayers of the four group-IV monochalcogenides. Herein, we applied new-model computational works predicting the hole mobilities to be 1 order of magnitude higher than the electron mobilities for GeSe.

In this study, we conducted a systematic analysis to estimate the deformation with defects, which transform circular Fermi surfaces to geometric forms, i.e., triangular and quadrangle forms, by applying an external potential. In this way, we obtain an exact solution for the deformation by performing an analytical method. First, the effective low-energy Hamiltonian is expressed by the tight-binding parameters, and the geometrical potentials are expressed by complying with the effective model. Then, structural and electronic parameters were analytically studied on the basis of modified-DPT theory .

## 2 Theoretical Model

**Figure 1:**
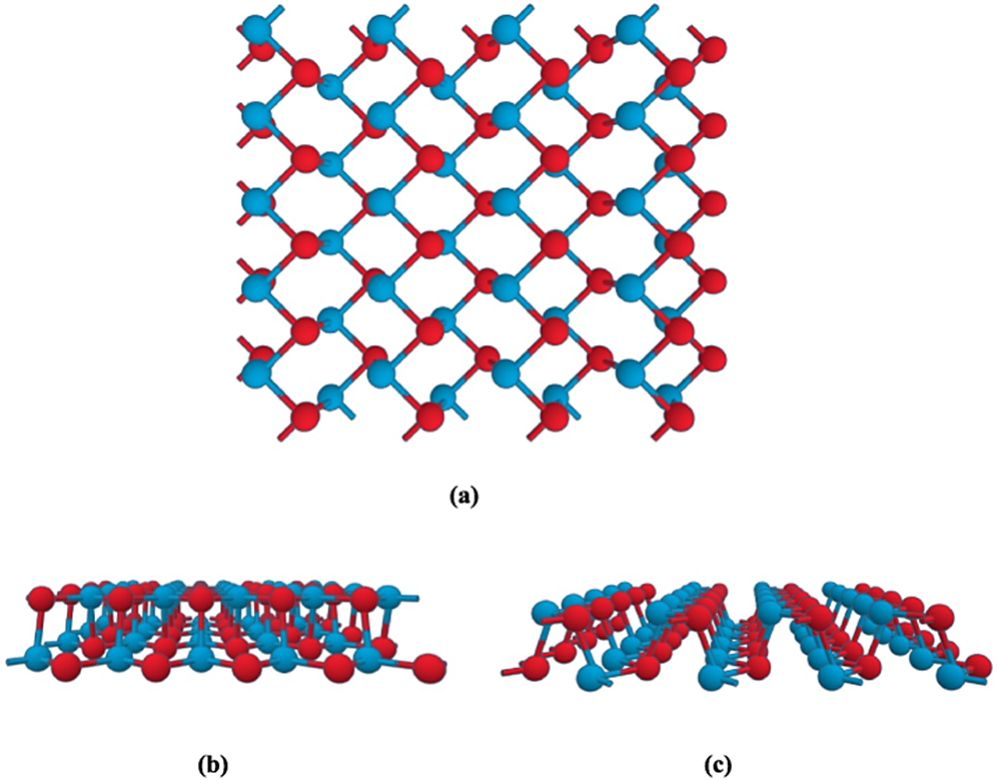
(a) Top view of a GeSe monolayer representing a group-IV monochalcogenide monolayer and showing the lattice along the *x*- and *y*-directions, (b) side view showing the *y*–*z* planes, and (c) side view showing the *x*–*z* planes.

The model discusses the low-energy bands of monochalcogenides around the Gamma (Γ)-point. It considers a particle moving at a geometrical potential that affects the Fermi surface topology of a material. The explicit effective low-energy model with a direct band gap can be represented as unperturbed parts that are close to the band edge and the effective models with direct band gap described in the form of Hamiltonians,
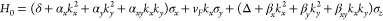
1where σ_*i*_ are the Pauli matrices and *k* = (*k*_*x*_,*k*_*y*_) are the *x* and *y* in-plane wave vectors. α_*i*_ and β_*i*_ specific values of the tight-binding parameters for GeSe have been obtained by fitting a known ab initio calculation.^37^ In this study, the coefficients of the low-energy model near the band edge are also used as those by Cook. Note that we used the restrictions α_*xy*_ = β_*xy*_ = 0, so that both conduction and valence bands are even under the symmetry. In this condition, the energy dispersion of the Hamiltonian in eq 1 is calculated as,
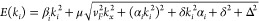
with *i* = (*x,y*). Herein, the term with the *v*_F_ Fermi velocity coefficient is the anisotropic term in the energy dispersion for the Hamiltonian. Its value can be determined from its crystal geometry. In the effective model for GeSe, the values of *v*_F_ depend on the tight-binding parameters, which include the *t*_*i*_ hopping parameters as, *v*_F_ = 2α_*x*_(*t*_2_ – *t*_3_) + (*t*_1_ + 2*t*_2_ + 2*t*_3_)*x*_0_ with *t*_1_ = −2.33 eV, *t*_2_ = 0.61 eV, and *t*_3_ = 0.13 eV. Here, the other parameters α_*i*_ and β_*i*_ should be known that  and  are the lattice constants of GeSe which has anisotropic geometry. *x*_0_ is the distance between the closest neighbors which is known as 4.8 Å.^38^*k* = (*k*_*x*_,*k*_*y*_) are the *x* and *y* in-plane wave vectors. Dirac electrons travel along the *x*- and *y*-axes with *v*_*x*_ and *v*_*y*_ that corresponding velocities, respectively. It can also be seen from Figure 1 that the charge carriers along the *x*- and *y*-directions are not exactly the same, which means that there is a slight anisotropic characteristic. Figure 1a shows the top view of a GeSe monolayer representing a Group IV monochalcogenide monolayer and showing the lattice in the *x*- and *y*-directions. Figure 1b shows the lateral view showing the *y*–*z* planes (zigzag), and Figure 1c shows the lateral view showing the *x*–*z* planes (armchair). Some studies in the expression of the Hamiltonian α_*xy*_ = β_*xy*_ = β_*x*_ = β_*y*_ = 0 express a simple formula for the energy conversion of the BPVE (bulk photovoltaic effect), using the formula in the papers because β_*x*_ and β_*y*_ do not give any contribution to calculation of the shift current and ballistic currents. But in this study we take into its contribution to the energy eigenvalues of the structure and have calculated by using the its value  and  with *t*_11_ = 0.07 eV and *t*_22_ = −0.09 eV.

**Figure 2:**
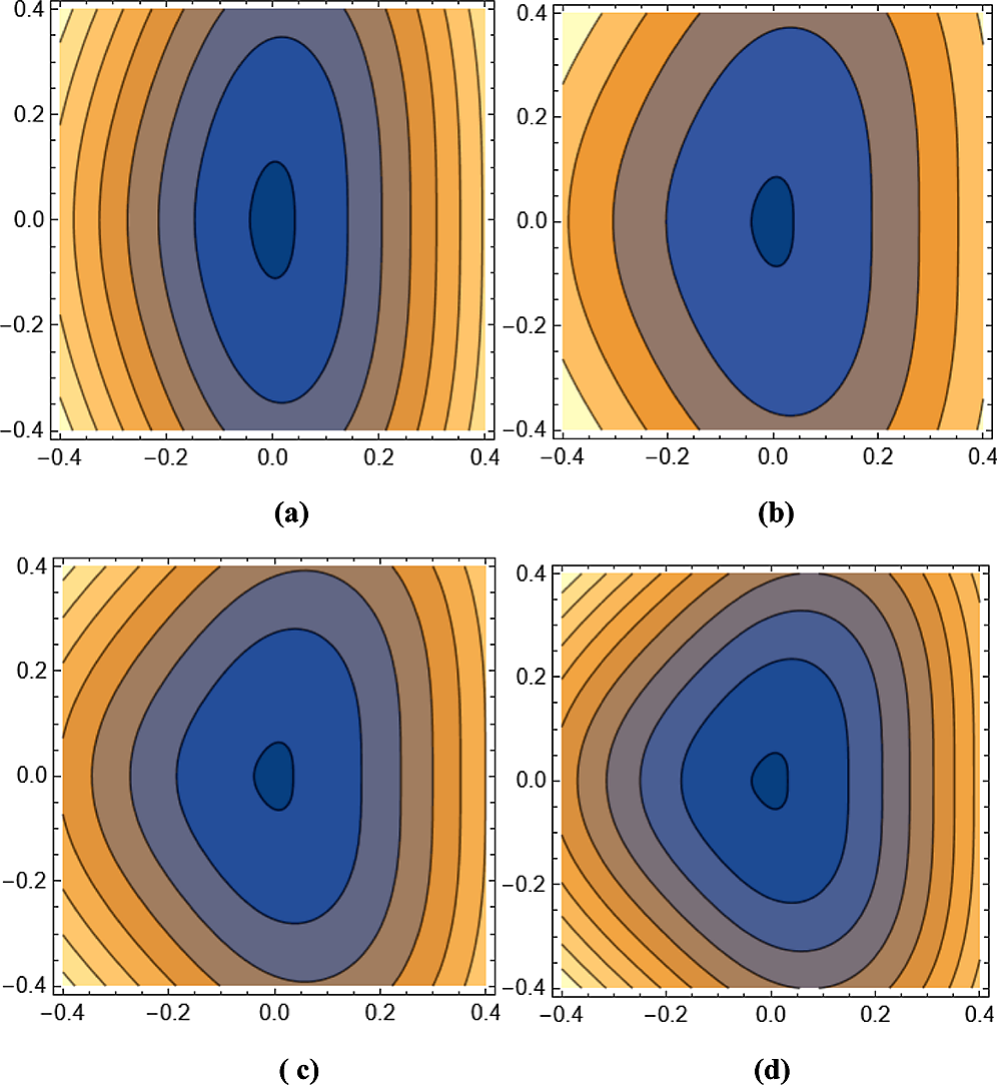
Fermi line topology which are close to the Γ point of the GeSe (a) *S* = 1, (b) *S* = 2, (c) *S* = 4, and (d) *S* = 6 respectively.

*H*_Q_ = σ_0_*S*_Q_*k* cos 4θ and *H*_T_ = σ_0_*S*_T_*k* cos 3θ are the geometrical potentials in GeSe. Here, σ_0_ is the identity matrix in the expression. *S*_Q_ and *S*_T_ are the constant parameters representing the quadrangular and triangular potential strength terms, so their values can increase the effectiveness of the potential properties in the system, respectively. The specific potentials modify the Fermi line topology of the structure, so that the circular contour plot of the GeSe evolves into triangular and quadrangular shapes. Thus, under the potential, the effective Hamiltonian is as follows:
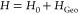
2where *H*_Geo_ is represented by *H*_Q_ or *H*_T_ depending on the Fermi surface topology. Contour plots of the potential were used to modify the trigonal and quadrilateral Fermi surface geometries on GeSe as shown in Figure 2. This figure shows how the Fermi line topology of GeSe near the point Γ changes with increasing geometric strength. As we increase the geometric strength, the circular form of the Fermi surface changes to a triangular structure. Figure 2 shows just an example of the transition from a circular to a triangular form and also shows the importance of geometric violence. The degree of violence and its effects will also be discussed in the electronic structure analysis.

The momentum dependent of the geometric terms can be given clearly for triangular and quadratic potentials respectively as,
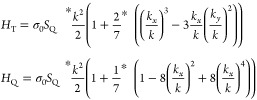
3where σ_0_ is the 2 × 2 identity matrix and *S*_Q_ is a constant parameter, representing the strength for the potentials. The strength term increases the effectiveness of the geometrical potential properties through the system. The change in potential modifies the Fermi line topology of the structure, so that the circular contour plot structure evolves into the other geometrical model. Thus, the geometrical potential-dressed system changes the Fermi line topology of the material and Fermi velocity. Additionally, the geometric potentials modify the effective mass and carrier mobility. The author demonstrates that the changes in FS topology, specifically electron topological transitions, are closely linked to transport behaviors at low temperatures.^39^ Additionally, theoretical and experimental investigations suggest that the magnetic field *H* and chemical potential ζ tuned by chemical doping could cause changes in the FS topology in topological materials.^39−41^

In this study, an analogue model with different strengths or increasing strength is used to determine the Fermi surface topology and physical parameters. Thus, the momentum dependent expression in the geometric part of the Hamiltonian written as eq 3 and more explicit guidelines can be obtained by considering a low-energy model with a geometrical part which is a direct band gap at the expanding Hamiltonian from eq 2. The carrier mobility of the 2D GeSe monolayer was computed together with the geometrical parts. The carrier mobility of the 2D GeSe monolayer was computed by many studies using the deformation potential (DP) theory, which was proposed by Bardeen and Shockley and has been successfully used to predict the carrier mobility of many 2D structures.^42^ Estimating the carrier mobility of 2D layered materials theoretically is challenging due to the involvement of multiple scattering processes, including charged-impurity, phonon, and Coulombic scattering.

The DP theory is an objective method for calculating carrier mobility, which can be expressed as,
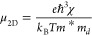
4

where *e* is the electron charge, *ℏ* is the reduced Planck constant, *k*_B_ is the Boltzmann constant, and *T* is the thermodynamic temperature. *X* = *C*_2D_/(*E*_1_)^2^ is the proportion of the elastic modulus and the square of the deformation potential in-plane stiffness, i.e., the elastic modulus *C*_2D_ obviously depends on the direction of the strain in the anisotropic structure. *E*_1_ represents the deformation potential (DP) constant, indicating the shift of the band edge (VBM for holes and CBM for electrons) caused by strain because of the defects. m* is the effective mass in the transport direction, and *m*_*d*_ is calculated as . The *m*_*x*_ and *m*_*y*_ are the effective masses along the armchair and zigzag directions of the carrier mass, respectively. Elastic modulus can be obtained through fitting the expression as (*E*–*E*_0_)/*S*_0_ = (*C*_2D_/2)(Δ*l*/*l*_0_)^2^, where *E* is the total energy and *S*_0_ is the equilibrium lattice volume of a 2D material. DP can be represented as, *E*_1_ = Δ*V*/(Δ*l*/*l*_0_), which is a crucial factor affecting the carrier mobility. Δ*V* represents the potential difference, and Δ*l*/*l*_0_ is the relative change in length. In mobility expression in eq 4 can be seen the definition of *X*. The expression shows the *X* = 2Δ*E*/*S*_0_(Δ*V*)^2^ explicit form. So we have no need to know strained and the equilibrium lattice parameters if we do not want to know the values of elastic modulus. Thus, by using the proportion of the elastic modulus, the deformation potential constant can be easily calculated for the single particle term to find the *X*.

## 3 Results and Discussion

In the monolayer structure, the carrier mobility, which is one of the most important properties of a semiconductor, along the *x-* and *y*-directions is not exactly equal, thus implying an anisotropic feature in the results. Mobility defines how quickly an electron or hole can drift in the material under an applied electric field, phonon contribution, or external potential. It is thus of primary importance for applications, such as FETs, photovoltaics, and thermoelectrics. It is known that tuning of the intrinsic properties of 2D materials is an essential step in the design of nanodevice applications. Because of this, temperature dependent changes have been studied. Temperature has a significant effect on the performance of semiconductor solar cells. As the temperature increases, the photovoltaic parameters such as the short-circuit current density, open-circuit voltage, fill factor, and power conversion efficiency generally decrease for most types of solar cells.^43^ The temperature effect on solar cell performances is attributed to the temperature-dependent band gap energy, charge carrier mobility, and lifetime. The charge carrier mobility has a significant influence on electron concentration, hole concentration, and Joule heat generation, while the charge carrier lifetime affects recombination heat generation.^43^ The efficiency of solar cells under concentrated illumination increases with concentration, but at a certain point, the negative effects of temperature increase suppress the positive effects of light concentration, leading to a decrease in efficiency. Overall, for understanding the temperature effects on carrier generation, we study on Figures 7 and 8. The calculation of carrier mobility based on geometrical potentials which are triangular and quadrangular, we take into account the electronic band structure along the symmetry directions of monolayer GeSe. The formula of the effective mass is . Therefore, in the presence of geometrical potential, the low-energy bands around the Gamma (Γ) point can be calculated by evaluating the band data in the Brillouin zone. As can be seen from Table 1, for monolayer GeSe, the effective mass of electrons is 0.24*m*_0_ for the *x*-direction and 2.53*m*_0_ for the *y*-direction, respectively. Also, the effective mass of holes is 0.27*m*_0_ along the *x*-direction and about 1.29 *m*_0_ along the *y*-direction. It can be seen that the effective mass in the *x*-direction is smaller than that in the *y*-direction, which indicates the anisotropic behavior along the armchair and zigzag directions. Some previous theoretical investigations on GeSe have shown that the electron effective mass is about 0.19–0.23*m*_0_ and about 0.37–0.46*m*_0_ along the *y*-direction. Hole effective mass is about 0.23–0.25*m*_0_ along the x-direction and about 0.59–0.87*m*_0_ along the *y*-direction,^44^ where *m*_0_ is the free electron mass. Comparing the undressed (without external effects) form with our geometric potential dressed form shows that the *x*-direction effects are dressed weak both for the electrons and holes. However, the geometric potential dressed along the *y*-direction is really heavy. Comparing the undressed effective mass with dressed results, a large amount of mass has been gained for both carriers that are electrons and holes along the *y*-axis.

**Table 1:** Effective Mass of Carriers with Geometrical Defects at *k*_*x*_ = *k*_*y*_ = 0.1 in the Gamma (Γ) Point of the Brillouin Zone

	carrier		
GeSe	electron	0.24	2.53
	hole	0.27	1.29

**Figure 3:**
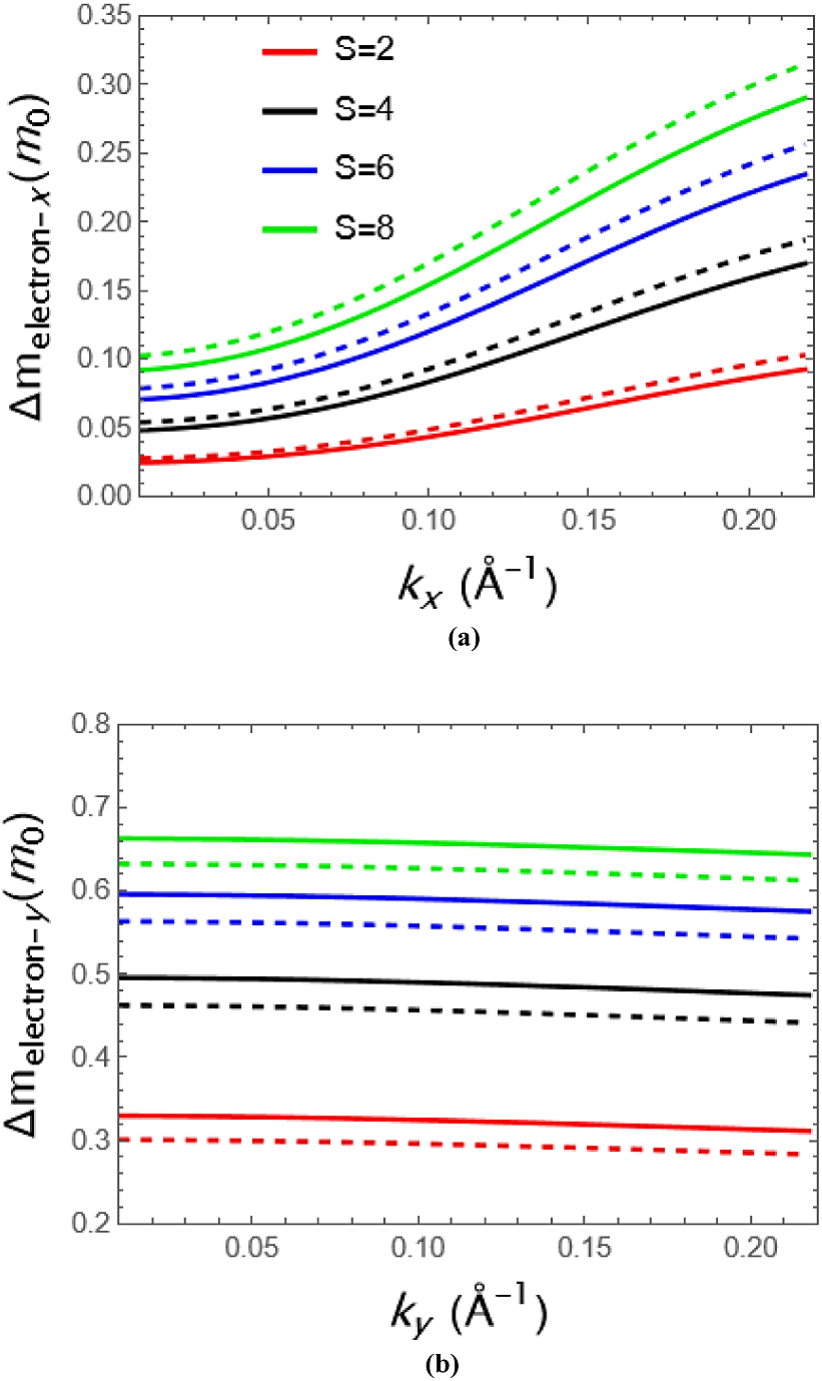
Electron mass behavior of GeSe as a function of (a) *k*_*x*_ and (b) *k*_*y*_ for different strengths.

**Figure 4:**
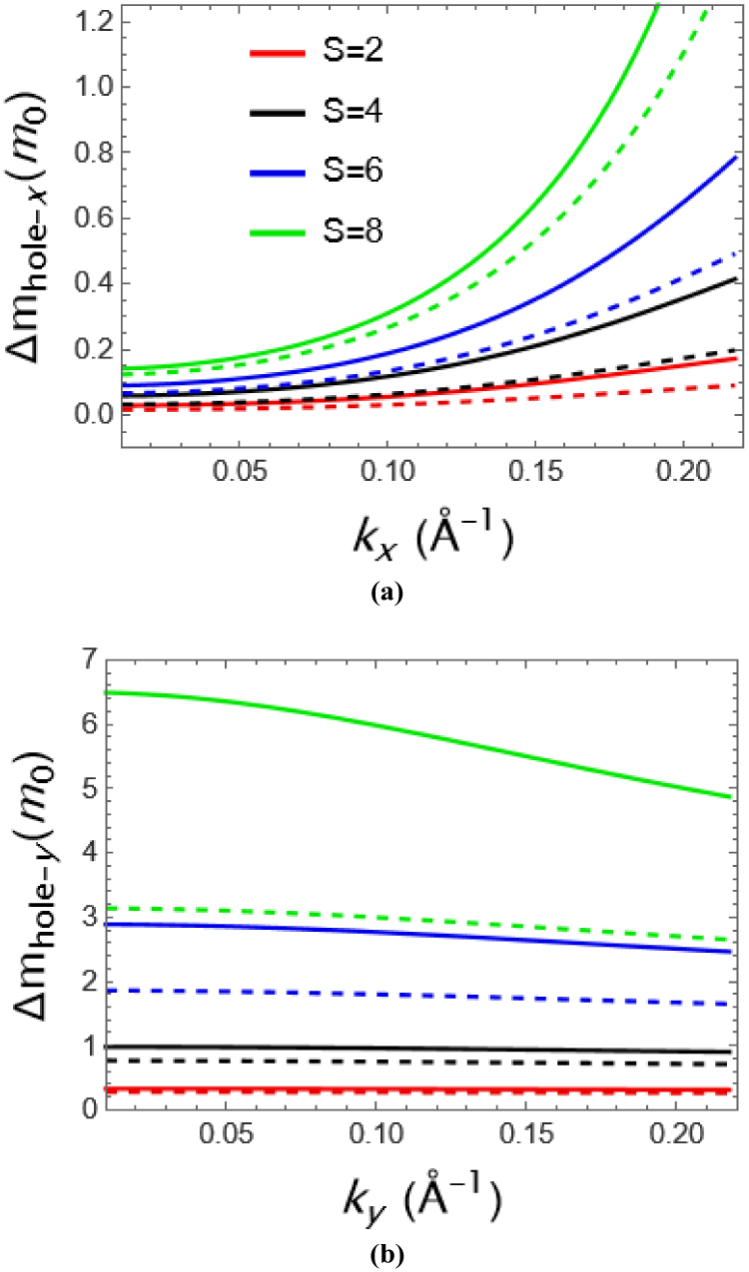
Hole mass behavior of GeSe as a function of (a) *k*_*x*_ and (b) *k*_*y*_ for different strengths.

In Figure 3, we first analyze the general dependence of the momentum and effective masses in GeSe. Herein, the effective mass renormalization of GeSe as a function of *k*_*x*_ and *k*_*y*_ can be seen explicitly and strongly depends on the strength for electrons. The effective masses are more marked in the zigzag direction that is along the *y*-direction while in the armchair direction, i.e., along the *x*-direction that its gaining mass is maximum %10 with strong strength. From Figure 3, straight lines represent the potential with quadratic geometry, while the dashed lines represent the triangular geometry for electrons. The increasing geometrical effects for both geometric potentials enhance the changes of effective masses through direction-dependent carriers in both directions. It also appears from the figure that the effective masses are decreasing slightly along the *y*-direction, but increasing strongly along the *x*-direction depend on the momentum for electrons. In Figure 4 geometrical effects on the hole effective masses along the *x*- and *y*-directions have been presented, and the straight lines represent the potential with quadratic geometry, while the dashed lines represent the triangular geometry. In this case, similar to Figure 3, the effective mass renormalization of the material as a function of momentum can be visible in a clear manner as shown in Figure 4. Nevertheless, it should be noted that the hole carrier gains more mass than the electron in both the *x*- and *y*-directions. In the *y*-direction, i.e., along the zigzag direction, the hole gains almost 10 times more mass than the electron. However, the momentum change in the *y*-direction is very fast, and the geometry of the potential used, i.e., whether it is triangular or quadriangular, also affects the effective mass value, especially at high strength values its value really different. Thus, it should be mentioned that in Figures 3 and 4, charge carriers have different effective masses due to the anisotropy of the model along the *x*- and *y*-directions.

**Figure 5:**
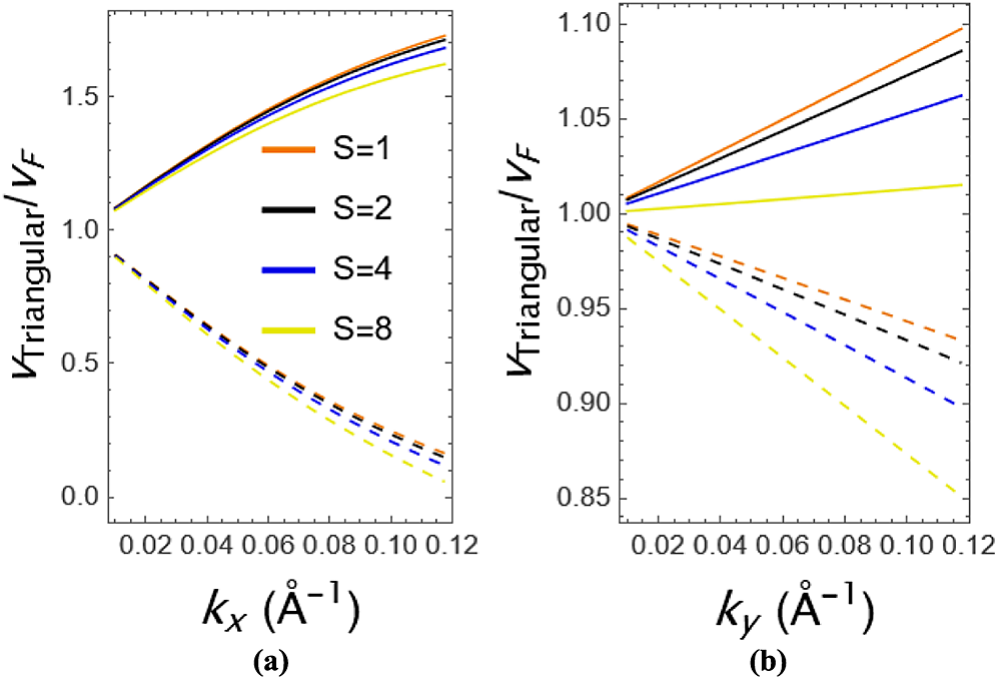
Velocity behavior of GeSe as a function of (a) *k*_*x*_ and (b) *k*_*y*_ for different strengths.

Effective velocities, ignoring the Berry curvature, the diagonal matrix elements of velocity can be calculated as , where *i* = *x* and *y* directions in the anisotropic structure. Figure 5 shows the Fermi velocity renormalization of GeSe under the triangular potential for the carriers of both, i.e., electrons and holes along the *x*- and *y*-directions. Straight lines are electrons; dashed lines are holes. The momentum dependence of the renormalization along the *x*-directional change is stronger than that of the *y*-directional change. When the momentum value comes to 0.12, its value reaches zero with holes along the *x*-direction but has a slight change along the *y*-direction. However, the value has a good value for electrons. Additionally, the strength-dependence of the behavior can be seen that the value increases with increasing strenght.

**Figure 6:**
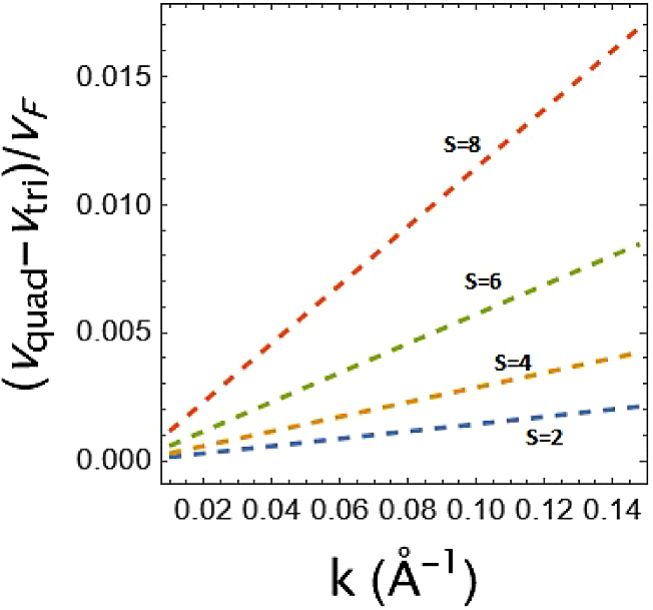
Effective velocity difference of the quadratic and triangular potentials.

**Figure 7:**
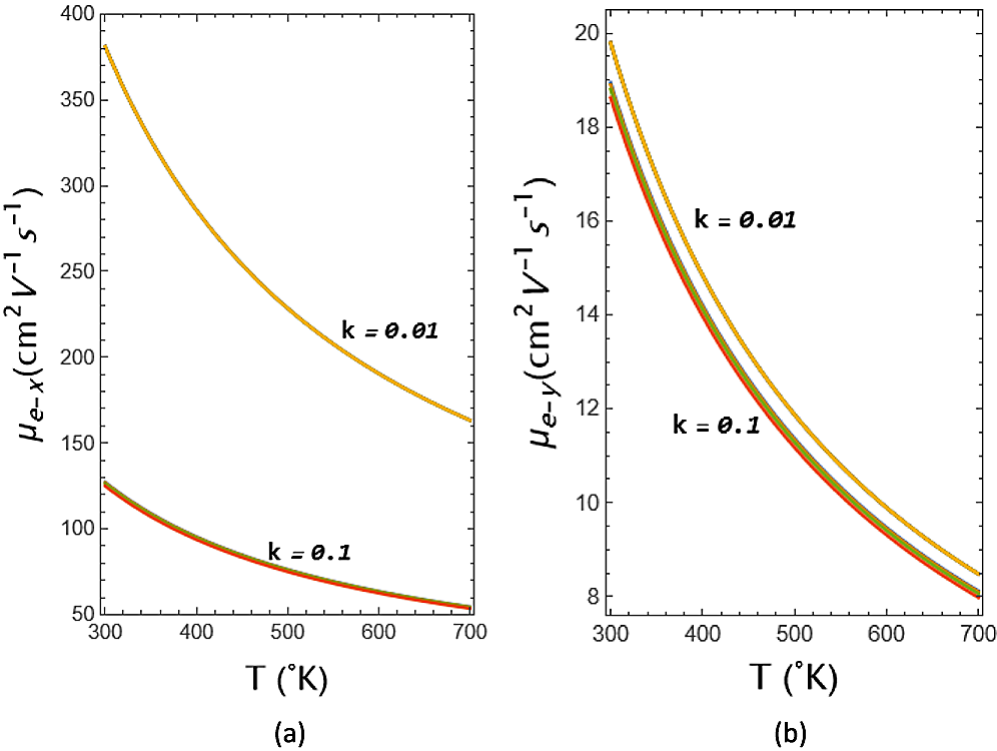
(a) *x*-dependence of the electron mobility of GeSe as a function of temperature at *k* = 0.01 and *k* = 0.1 for different strengths. (b) *y*-dependence of the electron mobility of GeSe as a function of temperature at *k* = 0.1 and *k* = 0.1 for different strengths.

**Figure 8:**
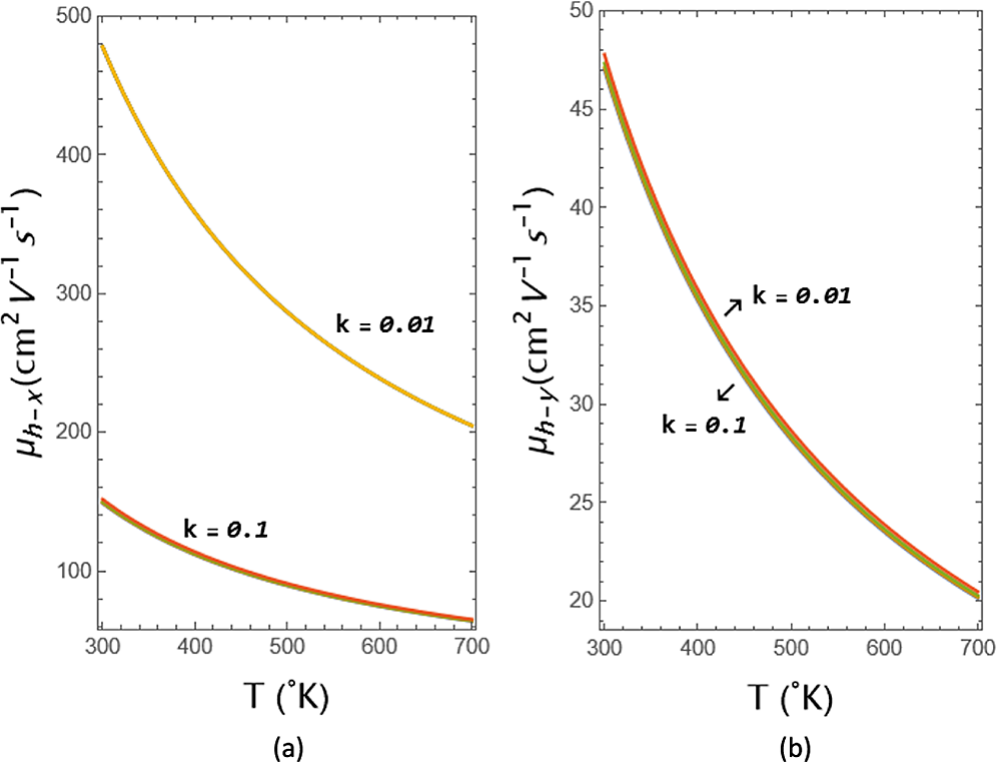
Coordinate dependence of the hole mobility of GeSe as a function of temperature for (a) *x*-axis and (b) *y*-axis for different strengths.

Another interesting property comes from Figure 6. The figure shows that there is no dependence of the geometrical potential used as a Fermi surface modifier at *k* = 0. But its value changes with increasing potential value. Moreover, this difference increases with increasing strength. When the strength value is 8, it is seen that the slope increases significantly. Figure 7a shows the *x*-dependence of the electron mobility of GeSe as a function of temperature. At room temperature, the value based on the geometrical potential modification is as high as 390 cm^2^ V^–1^ s^–1^ for electrons in *k* = 0.01, while 128 cm^2^ V^–1^ s^–1^ for the electrons in the *k* = 0.1 along the *x*-direction. In the figure, electron mobility of the *x*-axis component weakens considerably for *k* = 0.1 compared to thta for *k* = 0.01, with its value decreasing from 390 cm^2^ V^–1^ s^–1^ to 128 cm^2^ V^–1^ s^–1^. Additionally, from Figure 7a, the strength value for *k* = 0.1 is also visible hardly on the graph, even though it is very small, and it can be seen that the strength value has the lowest mobility value for *S =* 8, where the highest value of the strength value is taken. Although each of the figures plotted for the different strengths of the geometrical potential, for *k* = 0.01, all plots overlap and therefore not visible and even its difference can be seen hardly for *k* = 0.1 value. Figure 7b shows the *y*-dependence of the electron mobility of the monochalcogenide structure as a function of temperature at in *k =* 0.01, and in *k =* 0.1 for different strengths. The value based on the geometrical potential modification is really low, nearly 20 cm^2^ V^–1^ s^–1^ for *k* = 0.01 in the *y*-axis, while 19 cm^2^ V^–1^ s^–1^ for *k* = 0.1 in the *y*-axis. So, it can be seen the difference of *k* is not big subject for the electrons. Similarly, the figures plotted for the different strength of the geometrical potential. The small values of *k* all plots overlap, and from Figure 7b, it can be seen that they have just a small difference. Also, exhibiting a behavior similar to that in Figure 7a, Figure 7b shows the lowest mobility for the geometrical potential, which modifies the Fermi surface. Similarly, again, for *k* = 0.01, all the plots overlap and are therefore not visible, and even their difference is barely visible for *k* = 0.1 value. Figure 8a displays the *x*-dependence of the hole mobility of GeSe as a function of temperature. At 300°*K*, the value based on the geometrical potential modification is 480 cm^2^ V^–1^ s^–1^ for holes in *k* = 0.01 and 150 cm^2^ V^–1^ s^–1^ for holes in *k* = 0.1 with different strengths. In the figure, the hole mobility of the *x*-axis component is noticeably weakening for *k* = 0.1 compared to 0.01, with its value decreasing from 480 cm^2^ V^–1^ s^–1^ to 150 cm^2^ V^–1^ s^–1^. Figure 8b shows the *y*-dependence of the hole mobility of GeSe as a function of temperature. The mobility value under the potential is 55 cm^2^ V^–1^ s^–1^ for holes in *k* = 0.01, while it is 47 cm^2^ V^–1^ s^–1^ for holes in *k =* 0.1 with different strengths. In the figure, the hole mobility of the *y*-axis component weakens only slightly for *k* = 0.1 compared to that for *k* = 0.01, with its value decreasing from 55 cm^2^ V^–1^ s^–1^ to 47 cm^2^ V^–1^ s^–1^. In Figure 8a,b, similar to the Figure 7a,b, for a value of *k* = 0.01, all the plots overlap, making them invisible. Even their difference is barely noticeable for *k* = 0.1. In the mobility figures, it is seen that the contribution of the *y*-axis component mobility is very poor in the anisotropic structure. The primary contribution arises from the *x*-axis component, and temperature-dependent mobility can serve as a controllable parameter in GeSe. The very small contribution of strength has already been mentioned. Nevertheless, it should be noted that strength increases the mobility of holes and decreases the mobility of electrons.

## 4 Conclusion

In summary, the carrier mobility of GeSe is first investigated by a new analytical method based on the DPT theory. At room temperature (300 K), its changes with increasing temperature have been studied. The value based on the geometrical potential modification is as high as 390 cm^2^ V^–1^ s^–1^ (20 cm^2^ V^–1^ s^–1^) for electrons in the *x*-axis (*y*-axis), while 480 cm^2^ V^–1^ s^–1^ (54 cm^2^ V^–1^ s^–1^) for holes in the *x*-axis (*y*-axis) for *k* = 0.01. The results show that the electron mobility is mainly determined by the *x*-axis in the monochalcogenide structure, and the hole mobility is better than the electron on the structure. Then, we accurately calculate the mobility for *k* = 0.1. Comparing the results, it is found that the small values of the *k* are more effective for mobility both electrons and holes. The effective mass of monolayer GeSe in the *x*-direction is less than that in the *y*-direction. Consequently, the mobilities of electrons and holes in the *x*-direction are higher than those in the *y*-direction, indicating that GeSe exhibits obvious anisotropy. It is evident that the carriers are more readily transported in the armchair direction than those in the zigzag direction. We reveal that geometrical factors on the Fermi surface play a decisive role in the carrier mobility and Fermi velocity, similar to topological insulators.^45,46^ A comparison of the results also shows that there has been a large effect of temperature on the mobility. With increasing temperature, there is significantly reduced mobility that can be seen easily. The analysis indicated that the material exhibited a lower mobility rate in response to higher temperatures. On the other hand, the effective mass normalization value increases along the *x*-axis (armchair) but decreases along the *y*-axis with momenta (zigzag) for both electrons and holes in the dressed GeSe. However, in the anisotropic structure, the zigzag direction of the crystal plays an important role in the mass interactions so mass gaining. We analyzed the Fermi velocity effect of the dressed model; it is shown that the zigzag direction has a linear but armchair direction parabolic behavior. GeSe is a structure compatible with a p-type semiconductor. It is seen that in the GeSe monochalcogenide crystal, the hole mobility is higher than the electron mobility. In addition, the effective mass gain of holes is much higher than the effective mass gain of electrons. With such geometrical potential applications, its features as a p-type semiconductor are strengthened. The unique characteristics of n- and p-type semiconductors render them suitable for diverse applications in electronic devices. Conversely, p-type semiconductors are employed in devices such as solar cells, where the movement of holes is indispensable for the generation of an electric current. They are also employed in diodes and bipolar junction transistors, which necessitate a regulated flow of the electric current. From the advancement of transistors and diodes to the development of solar cells and thermoelectric devices, n- and p-type semiconductors continue to be pivotal in technological innovation. Thus, the materials are very promising for thermoelectric applications, and with altering its electronic properties, the material can be controlled for electronic applications.
